# Severe Thrombocytopenia in a Case of Epstein-Barr Virus-Induced Infectious Mononucleosis

**DOI:** 10.7759/cureus.17706

**Published:** 2021-09-04

**Authors:** Chen Zhang, Anne M Kelly

**Affiliations:** 1 Medicine, University of Cambridge School of Clinical Medicine, Cambridge, GBR; 2 Paediatric Haematology, Addenbrooke's Hospital, Cambridge, GBR

**Keywords:** paediatric haematology, thrombocytopenia, infectious mononucleosis, ebv, atypical lymphocyte, immune-mediated thrombocytopenia

## Abstract

Epstein-Barr virus (EBV) infections have variable presentations ranging from asymptomatic to the triad of fever, pharyngitis, and adenopathy in infectious mononucleosis. Although haematological abnormalities are commonly seen in EBV infections, severe EBV-associated thrombocytopenia is a rare presentation, complicating clinical diagnosis and requiring appropriate management. Here we describe a case of a 14-year-old female with severe thrombocytopenia (platelet count of 5 x10^9^/L) and spontaneous haemorrhage, accompanied by periorbital oedema, an uncommon symptom in EBV-associated infectious mononucleosis. She was treated with intravenous immunoglobulins and a four-day course of methylprednisolone. Treatment resulted in progressive platelet count recovery, and the patient was discharged seven days post-admission with a platelet count of 143 x10^9^/L. The case highlights the need to consider EBV infection as a differential diagnosis in patients presenting with acute severe thrombocytopenia.

## Introduction

Epstein-Barr virus (EBV) is an extremely common herpesvirus infecting approximately 90% of the world’s population before the age of 30 [1]. It is the leading cause of infectious mononucleosis (IM), with roughly 90% of IM cases caused by EBV and the remaining cases largely due to cytomegalovirus (CMV), human herpesvirus 6, toxoplasmosis, human immunodeficiency virus (HIV), and adenovirus [2].

IM classically presents as a triad of fever, pharyngitis and adenopathy with prodromal symptoms of fatigue, malaise and myalgia. A less common clinical feature is the ‘Hoagland Sign’, an early periorbital swelling which Hoagland reported in one-third of IM cases [3]. Additionally, mild-to-moderate thrombocytopenia occurs in 50% of uncomplicated cases of IM [4]. However, severe thrombocytopenia (platelet count < 20 x10^9^/L) is exceedingly rare [5]. A case of a 14-year-old girl with Hoagland’s sign and severe thrombocytopenia secondary to EBV-associated IM is reported.

## Case presentation

A previously well 14-year-old girl was admitted to the hospital after presenting with spontaneous bleeding from the scalp and mouth, diffuse petechial rash, and spontaneous bruising of the legs. She had been suffering from a two-week history of periorbital oedema, malaise, fatigue, and worsening throat pain. She was fully immunised and had no family history of bleeding disorders. On admission, she had normal vital signs. Physical examination revealed bleeding from the scalp, conjunctival haemorrhage, petechiae over the tongue and buccal mucosa, a diffuse petechial rash over the upper body, and bruising on both legs. Cervical lymphadenopathy and oedematous tonsils were also noted. Systemic examination was normal with no hepatosplenomegaly.

**Figure 1:**
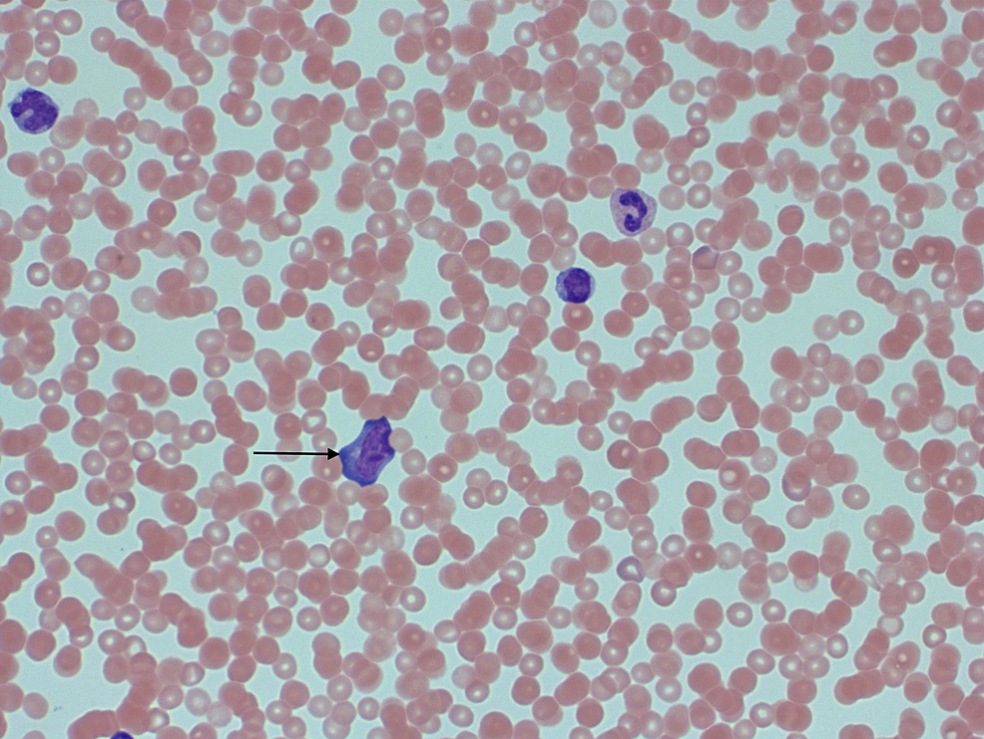
Blood film showing reactive lymphocytosis Blood film showing a reactive lymphocyte with the ‘Ballerina skirt’ feature, characterised by a basophilic cytoplasm moulding around adjacent erythrocytes.

Initial laboratory investigations revealed that the patient was severely thrombocytopenic with a platelet count of 5 x10^9^/L. Her prothrombin time and activated partial thromboplastin time were prolonged at 14.1 s and 37.1 s, respectively. She had normal haemoglobin 119 g/L and a total white cell count of 10 x10^9^/L (elevated lymphocytes 5.11 x10^9^/L with atypical lymphocytes). A peripheral blood smear was highly suggestive of acute EBV infection and showed reactive lymphocytosis and thrombocytopenia (Figure 1). Immunophenotyping subsequently confirmed a CD8+ T cell lymphocytosis of 3.2 x10^9^/L with human leukocyte antigen DR (HLA-DR) expression suggestive of a viral infection. Lactate dehydrogenase was raised at 425 units/L, and liver function tests showed elevated alanine transaminase (ALT) at 135 units/L. Renal function test and urine examination were normal.

Intravenous methylprednisolone, oral tranexamic acid, and 1 g/kg intravenous immunoglobulins (IVIG) were started to treat the bleeding diathesis along with lansoprazole for gastric protection. The IVIG therapy was complicated by an adverse reaction in the form of fever, chills, flushing of the face, an erythematous rash on the left arm, lethargy, and vomiting, which gradually subsided. She was discharged seven days after admission with a platelet count of 143 x10^9^/L.

A positive EBV serology was seen with the presence of both viral capsid antigen immunoglobulin M (IgM) and immunoglobulin G (IgG). EBV-DNA was detected by a polymerase chain reaction in the peripheral blood. Additionally, IgG antibodies to Epstein-Barr nuclear antigen were negative, and CMV and adenovirus DNA were not detected. The patient was diagnosed with immune-mediated thrombocytopenia secondary to an acute EBV infection.

## Discussion

Various theories have been proposed to describe the pathophysiology of EBV-induced thrombocytopenia. One hypothesis is the activation of a viral-induced autoimmune response resulting in the formation of antibodies targeting platelet glycoproteins IIb-IIIa and Ib-IX. However, antiplatelet antibodies were only detected in 40% of patients with an EBV infection [6]. An alternative theory is the splenic sequestration of platelets secondary to hypersplenism. However, many patients with splenomegaly have normal platelet counts, and patients with serious thrombocytopenia can have normal-sized spleens, as is the case with our patient. Other theories include vascular damage, hemophagocytic lymphohistiocytosis and EBV presence in the bone marrow, impairing platelet generation [7].

Individual case reports of severe thrombocytopenia in patients with EBV infections have shown varied platelet recovery times. While some reports show thrombocytopenia lasting weeks [8] and the rare development of chronic thrombocytopenia [9], others report spontaneous platelet count recovery within days [10]. Although the case presented here shows rapid platelet count recovery following treatment, the need for specific therapy has been questioned.

The National Institute for Health and Care Excellence (NICE) recommended first-line treatment options for acute immune thrombocytopenic purpura (ITP) includes corticosteroids and IVIG. Platelet transfusions offer temporary benefits, with their use reserved for controlling severe life-threatening haemorrhage. There is some evidence that IVIG infusion preceding platelet transfusion may increase platelet survival via its immunomodulatory mechanisms [11]. Reports have suggested that patients with EBV-induced severe thrombocytopenia are often refractory to corticosteroid therapy, with cases showing slow rises in patients' platelet counts with or without steroid treatment [12,13]. Alternatively, high dose IVIG was shown to rapidly increase platelet counts [14]. However, IVIG adverse effects can occur, including an immediate flu-like response involving fever, headache, vomiting and dyspnoea. It has been suggested that these immediate side effects occur due to the presence of IgG aggregates in IVIG preparations triggering complement activation [15]. Reduced IVIG infusion rates and pre-infusion treatment with analgesics and non-steroidal anti-inflammatories have been shown to reduce immediate adverse effects of IVIG [16].

In addition to thrombocytopenia, EBV-associated IM can result in other haematological abnormalities, including atypical lymphocytosis (AL). AL is characterised by an increased number of large reactive lymphoid cells on a Wright-stained peripheral smear. Although AL is not specific to EBV infections, it has been shown to have a particularly close association with EBV-related IM. Along with AL, elevated lactate dehydrogenase (LDH) and gamma-glutamyltransferase (GGT) indicating hepatocellular damage have also been proposed to help differentiate EBV-associated IM from CMV-associated IM [17]. The patient, in this case, showed AL with elevated LDH and alanine aminotransferase (ALT) but a normal GGT.

The patient in this case also presented with periorbital oedema, a symptom that has been described to be more specific to EBV-associated IM. It is thought that nasopharyngeal replication of EBV causes abnormal orbital lymphatic drainage, which results in periorbital swelling [18]. First described by Hoagland in 1952, this less well-known clinical manifestation often occurs as an early finding and can help clinicians in the diagnosis of EBV-associated IM [19].

## Conclusions

EBV-associated IM typically presents as a triad of fever, pharyngitis and lymphadenopathy accompanied by atypical lymphocytosis. However, there is an occasional occurrence of less common features including periorbital oedema and severe thrombocytopenia. This case report highlights the need to be aware of these features in order to aid clinical diagnosis and provide appropriate management of the bleeding diathesis.
